# Potentials of Long Noncoding RNAs (LncRNAs) in Sarcoma: From Biomarkers to Therapeutic Targets

**DOI:** 10.3390/ijms18040731

**Published:** 2017-03-29

**Authors:** Li Min, Cassandra Garbutt, Chongqi Tu, Francis Hornicek, Zhenfeng Duan

**Affiliations:** 1 Sarcoma Biology Laboratory, Department of Orthopaedic Surgery, Massachusetts General Hospital and Harvard Medical School, 55 Fruit Street, Jackson 1115, Boston, MA 02114, USA; jacky-min@163.com (L.M.); Cassandra.Garbutt@mgh.harvard.edu (C.G.); FHORNICEK@mgh.harvard.edu (F.H.); 2 Department of Orthopedics, West China Hospital, Sichuan University, 37 Guoxue Road, Chengdu 610041, Sichuan, China; tuchongqi@163.com

**Keywords:** long noncoding RNA, sarcoma, metastasis, prognosis

## Abstract

Sarcoma includes some of the most heterogeneous tumors, which make the diagnosis, prognosis and treatment of these rare yet diverse neoplasms especially challenging. Long noncoding RNAs (lncRNAs) are important regulators of cancer initiation and progression, which implies their potential as neoteric prognostic and diagnostic markers in cancer, including sarcoma. A relationship between lncRNAs and sarcoma pathogenesis and progression is emerging. Recent studies demonstrate that lncRNAs influence sarcoma cell proliferation, metastasis, and drug resistance. Additionally, lncRNA expression profiles are predictive of sarcoma prognosis. In this review, we summarize contemporary advances in the research of lncRNA biogenesis and functions in sarcoma. We also highlight the potential for lncRNAs to become innovative diagnostic and prognostic biomarkers as well as therapeutic targets in sarcoma.

## 1. Introduction

Sarcoma is a heterogeneous group with more than 70 malignant primary neoplasms of mesenchymal origin [1,2]. The most common sarcomas originate in the bone and soft tissue, which account for 19–21% of all cancer-related deaths in children and adolescents [2,3]. At present, the diagnosis of a sarcoma is dependent on the clinical description, radiographic assessment, and histopathologic staging systems. The standard treatment regimen includes surgery combined with neoadjuvant chemotherapy and irradiation therapy. At inception 30 years ago, standard treatment enhanced disease prognosis, but no innovations in treatment strategy have changed prognosis since then [2]. However, new approaches in treatment are needed, as patients with metastatic or recurrent disease have an overall survival rate of less than 20% [4]. To improve diagnosis, treatment and prognosis, an enriched understanding of the genetic and epigenetic changes associated with sarcoma onset and progression is essential for biomarker and therapeutic target identification. Over the past two decades, knowledge of sarcoma molecular pathogenesis has grown without translation into effective methods for diagnosis and prognosis.

Most previous genetic studies are focused on the genes of protein coding RNAs [5,6,7,8,9]. However, protein coding RNAs account for only 2% of the human transcriptome [10,11,12,13,14,15]. The majority of human genome transcripts are non-coding RNAs (ncRNA) which are not transcriptional noise as previously believed. In fact, a number of ncRNAs have been described as direct or indirect influencers of gene expression [10,11,12,13,14,15]. A recently popular area of cancer biology research pertains to the investigation of ncRNAs involved in tumorigenesis. NcRNAs are generally classified into two groups according to their length, namely small ncRNAs (sncRNAs) and long noncoding RNAs (lncRNAs). SncRNAs are less than 200 nucleotides and include microRNAs (miRs), Piwi-interacting RNA (piRNAs), transcription initiation RNAs (tiRNAs), endogenous small interfering RNAs (endo-siRNA) and others [16,17,18].

LncRNAs are linear RNA transcripts, are more than 200 nucleotides long, maintain no protein coding potential, and are highly abundant and heterogeneous. In comparison to protein coding transcripts, lncRNAs have higher tissue specificity, lower expression levels, and less conservation on a sequence level [19,20,21]. Compared with sncRNAs, the functions and mechanisms of lncRNAs are poorly understood. The number of human lncRNAs identified is growing past the current catalog of more than 56,000 transcripts [22,23]. In eukaryotic cells, a number of lncRNAs have been shown to regulate DNA modifications, RNA transcription, pre-mRNA splicing, mRNA degradation, and mRNA translation, in addition to other functions that influence genetic and epigenetic expression levels [24,25]. Functions of many lncRNAs are linked with cancers that nonexclusively include those originating in the colon, lung, breast, stomach, and connective tissue [26,27,28,29,30]. Dysregulation of lncRNAs can influence metastasis, chemosensitivity, and tumor cell growth and proliferation [31,32,33,34].

Recently, in sarcoma research, a variety of lncRNAs have been considered for their potential as therapeutic targets and biomarkers for diagnosis and prognosis. The detailed mechanisms of lncRNAs in sarcoma remain under investigation. In this review, we summarize recent studies on sarcoma-related lncRNAs and discuss their potential relevance for innovating diagnostic, treatment and prognostic approaches.

## 2. Classification and Functions of Long Noncoding RNAs (LncRNAs)

**Figure 1:**
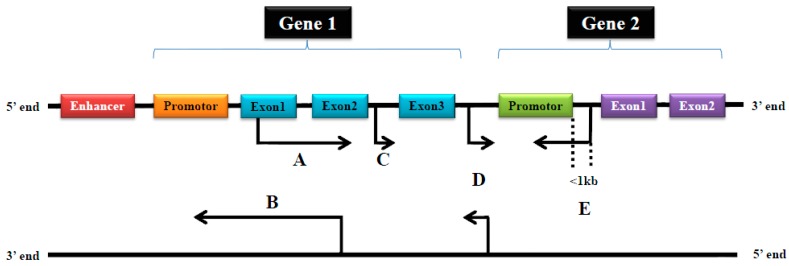
The classification of long noncoding RNAs (lncRNAs). (**A**) Sense lncRNAs are transcribed from the sense strand of protein-coding genes. They contain exons from protein-coding genes and therefore can share a part or the entire sequence of a protein-coding gene; (**B**) Antisense lncRNAs are transcribed from the antisense strand of protein-coding genes and they contain exons from protein-coding genes, and therefore can share a part or the entire sequence of a protein-coding gene; (**C**) Intronic lncRNAs are transcribed from an intron and therefore contain a sequence originating from the genomic interval between two exons; (**D**) Intergenic lncRNAs are transcribed from both strands and are an independent unit within the genomic interval between two genes; and (**E**) Bidirectional lncRNAs are transcribed from the promoter of a protein-coding gene in opposite direction of the protein-coding gene. Their sequence is located less than 1kb upstream from the promoter sequence.

According to their relationship with protein-coding genes, lncRNAs are classifiable into five main categories: sense, antisense, intergenic, intronic and bidirectional lncRNAs [27] (Figure 1). Sense or antisense lncRNAs overlap with at least one exon of another transcript from the same or opposite strand, respectively [35,36]. Intergenic lncRNAs are within a genomic interval in between two coding genes that are at least 1000 base pairs away from the nearest coding gene [35,36,37]. Intronic lncRNAs initiate inside an intron in either direction and terminate without overlapping exons [35,36]. Bidirectional lncRNAs are on the complementary strand of protein-coding genes and they are less than 1000 base pairs downstream from the transcription initiation site [35,36].

**Figure 2:**
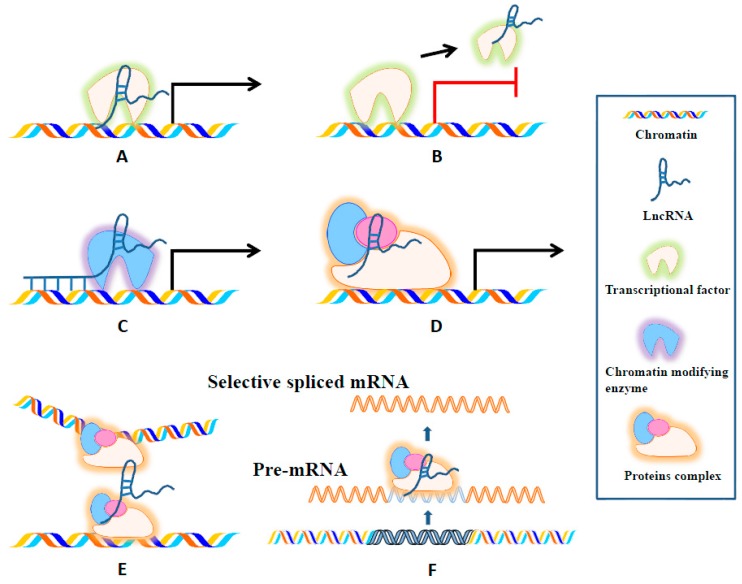
Schematic diagram of the six mechanisms of lncRNA functioning at transcriptional level. (**A**) LncRNAs can signal the activation of gene expression by integrating with one or more transcriptional factors; (**B**) LncRNAs can remove transcriptional factors from chromatin or they can compete for the miRNA target site, and, if successful, both mechanisms hault gene expression; (**C**) LncRNAs bind with chromatin, and this unit recruits chromatin modifying enzymes before guiding them to a target site for the initiation of gene expression; (**D**) LncRNAs can act as scaffolds to bring together multiple proteins and form ribonucleoprotein complexes, which subsequently induce gene expression; (**E**) LncRNAs regulate gene expression by acting on the spatial conformation of chromosomes; and (**F**) LncRNAs can hybridize to pre-mRNAs, block spliceosome recognition of splice sites, and regulate the alternative splicing of pre-mRNAs to produce different transcripts.

**Figure 3:**
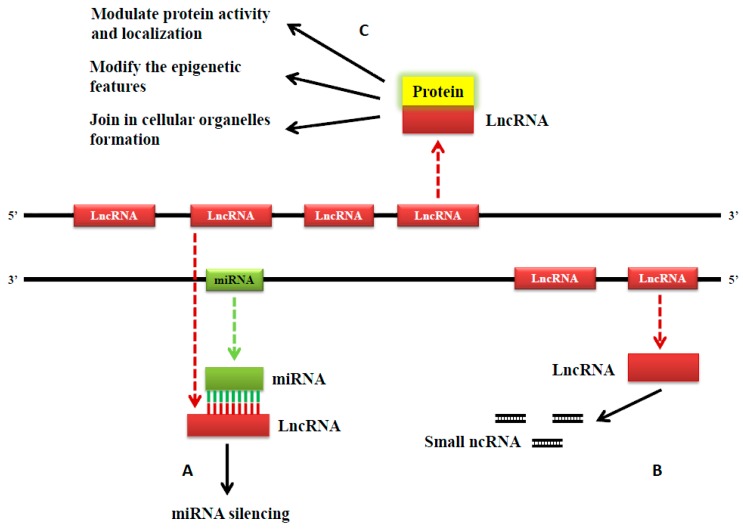
Schematic illustration of lncRNAs functioning at a post-transcriptional and epigenetic level. (**A**) LncRNA can silence miRNA by binding to it; (**B**) LncRNA can be precursors of sncRNA; and (**C**) When interacting with proteins, lncRNAs are capable of the following: modulating protein activity and localization; modifying epigenetic features including DNA methylation, histone methylation, acetylation or ubiquitination, and more; and contributing to the formation of cellular organelles.

LncRNAs can modulate protein-coding gene expression in several ways to influence cell proliferation, metastasis, drug resistance and more. They can interact directly with DNA, mRNA, and proteins to regulate a plethora of mechanisms including: chromatin modifications, transcription, splicing, and translation [10,35,38]. Firstly, lncRNAs can promote or suppress gene expression at the transcriptional level (Figure 2). When lncRNAs induce transcription, they act as enhancer signals or guides for recruiting chromatin modifying complexes, but lncRNAs can also suppress transcription when they act as a decoy and directly bind to a transcription factor, which effectively prevents the transcription factor from binding with its target gene promoter [39,40]. LncRNAs can also regulate gene expression by influencing the spatial conformation of chromosomes [41]. Moreover, lncRNAs can hybridize to pre-mRNAs, block spliceosome recognition of splice sites, and regulate the alternative splicing of pre-mRNAs to produce different transcripts [42]. Additionally, lncRNAs can act as a scaffold by integrating RNA binding proteins that affect the target gene’s transcription [43]. LncRNAs also exert functions at the post-transcriptional level (Figure 3). They protect target mRNAs from degradation by forming double-stranded RNA complexes [44,45]. Moreover, some sncRNAs are processed from lncRNAs [46]. Lastly, by modulating protein activity and localization, lncRNAs influence epigenetic features including DNA and histone methylation, acetylation, ubiquitination, and more. LncRNAs also contribute to cellular organelle formation [25,47,48,49] (Figure 3).

## 3. The Functions and Mechanisms of LncRNAs in Osteosarcoma

Osteosarcoma is the most common primary malignancy of bones and the third most common primary malignancy in children and adolescents [50,51]. Due to the extremely heterogeneous and complex nature of the origin and development of osteosarcoma, early diagnosis is an immense challenge. Surgery combined with neoadjuvant chemotherapy is the current standard treatment for osteosarcoma; however, patients with metastasis, recurrence and chemoresistance have poor outcomes [2]. Increasing evidence demonstrates that lncRNAs regulate cell growth and proliferation in osteseosarcoma, and dysregulation of lncRNAs is related to patient outcome including prognosis, metastasis, and recurrence.

### 3.1. LncRNAs in Osteosarcoma Cell Proliferation

Some lncRNAs can affect osteosarcoma cell proliferation through specific pathways. In both osteosarcoma tissues of human primary and mouse xenograft origins, H19 was highly upregulated [52]. Mechanistically, H19 is positively influenced by the hedgehog signaling pathway and the oncogene yes-associated protein 1 (Yap1) [52]. Decreased human osteosarcoma cell viability and downregulated H19 occurs after the inhibition of the hedgehog signaling pathway or knockdown of the *Yap1* gene [52]. Metastasis-associated lung adenocarcinoma transcript 1 (MALAT1) was found to be highly expressed in human osteosarcoma tissue [53]. Knockdown of MALAT1 inhibited the proliferation of human osteosarcoma cell lines via the phosphatidylinositol 3-kinase (PI3K)/Akt signaling pathway [53]. On the other hand, BRAF-regulated lncRNA 1 (BANCR) was extremely low in osteosarcoma MG-63 cells [54]. BANCR overexpression reduced cell proliferation via the upregulation of phosphorylated c-Jun N-terminal kinases (JNK) and downregulation of the Wnt/β-catenin signaling pathway [54] (Table 1).

Other lncRNAs affect osteosarcoma cell proliferation by interacting with miRNAs and lncRNAs. The expression levels of taurine upregulated gene 1 (TUG1) were significantly higher in human osteosarcoma tissue compared with matched non-tumorous tissue [55]. TUG1 suppression by siRNA significantly reduced cell proliferation in human osteosarcoma cell lines by apoptosis induction [55]. Further research confirmed the results, and indicated that TUG1 downregulation was capable of decreasing tumor growth in vivo [56]. The target of miR-9-5p is 3′UTR of POU2F1 [56]. TUG1 can upregulate POU2F1 expression and downregulate miR-9-5p expression by directly sponging miR-9-5p [56]. ZEB1-AS1 Zinc Finger E-Box Binding Homeobox 1 Antisense RNA 1 (ZEB1-AS1) expression was upregulated in human osteosarcoma tissue and cell lines [74]. The overexpression of ZEB1-AS1 was correlated with a larger tumor size and progressed Enneking staging in osteosarcoma patients [74]. The upregulation of ZEB1-AS1 promoted osteosarcoma cell proliferation while ZEB1-AS1 knockdown expectedly inhibited osteosarcoma cell proliferation [74]. ZEB1-AS1 directly binds and recruits p300 to the ZEB1 promoter region, induces an open chromatin structure, and activates ZEB1 transcription [74]. This mechanism related to ZEB1-AS1 knockdown suggests a significant correlation between the expression of ZEB1-AS1 and ZEB1 in human osteosarcoma tissue [74]. The downregulation of ZEB1 inhibited the roles of ZEB1-AS1 in osteosarcoma cell proliferation [74]. Other lncRNAs can also influence the proliferation of osteosarcoma cells by interacting with different ncRNAs (Table 1).

LncRNAs can influence osteosarcoma cell proliferation by other mechanisms, such as controlling gene expression. MALAT1 knockdown remarkably reduced the formation of tubular network structures and caused the breakage of stress fibers in human osteosarcoma cell lines [59]. Specifically, the downregulation of MALAT1 depressed protein levels of RhoA and its downstream effectors, Rho-associated coiled-coil containing protein kinases (ROCKs) [59]. In osteosarcoma samples, homeobox (HOX) transcript antisense RNA (HOTAIR) was commonly over-expressed as well as correlated with advanced tumor stage and high histological grade [60,61]. The downregulation of HOTAIR notably suppressed cellular proliferation by inhibiting matrix metalloproteinase 2 (MMP2) and MMP9 [60]. In another study, the inhibition of HOTAIR downregulated the transforming growth factor-β (TGF-β) and Bcl-2, and also upregulated p53 and the tumor necrosis factor-α (TNF-α) [61] (Table 1).

### 3.2. LncRNAs in Osteosarcoma Diagnosis

Osteosarcoma is driven by transcriptional deregulation, abnormal kinase signaling, epigenetic reprogramming and more [75]. So far, there are no specific molecular diagnostic markers [75]. LncRNAs are verified as important regulators of transcription. Recent studies have identified candidate lncRNAs as potential diagnostic biomarkers of osteosarcoma.

After microarray analysis of 25,733 lncRNAs in osteosarcoma, 403 lncRNAs were upregulated and 798 lncRNAs were under-regulated [76]. Bioinformatic analysis (gene ontology analysis, pathway analysis and network analysis) revealed that 32 pathways corresponded with under-regulated transcripts and 34 pathways corresponded with over-regulated transcripts [76]. These differentially expressed lncRNAs in osteosarcoma are potential candidates for novel diagnostic biomarkers [76]. Compared with normal tissue, the expression level of HOTAIR was significantly higher in osteosarcoma patients [77]. The C allele of single nucleotide polymorphism (SNP) rs7958904 from HOTAIR was associated with a decreased risk for osteosarcoma development when compared with the G allele [77]. Functional analyses on HOTAIR expression showed that the SNP rs7958904 CC genotype had significantly lower HOTAIR RNA levels than those of other genotypes [77]. The SNP rs7958904 CC genotype may negatively affect the risk for osteosarcoma development [77]. Therefore, the C allele of SNP rs7958904 of HOTAIR may be a new diagnostic biomarker for osteosarcoma. In blood samples of osteosarcoma patients, TUG1 expression levels were decreased in postoperative patients in comparison with preoperative patients, and the changes of TUG1 expression were significantly associated with disease status [78]. Moreover, TUG1 expression was successful in differentiating osteosarcoma patients from healthy individuals [78].

### 3.3. LncRNAs in Osteosarcoma Metastasis

As one of the hallmarks of malignancy, metastasis is a complicated process. The five-year survival rate for patients with localized disease is 80%. However, the prognosis is poor for those with metastatic osteosarcoma [79]. Emerging evidence has revealed a close relationship between lncRNAs and osteosarcoma metastasis. As a side-note, in human breast tumors, HOTAIR is correlated with metastasis [80].

Some lncRNAs act in different pathways to influence osteosarcoma cell invasion and migration. The level of MALAT1 was closely correlated with pulmonary metastasis in human osteosarcoma tissue [53]. In human osteosarcoma cell lines, the knockdown of MALAT1 by siRNA affected the PI3K/AT signaling pathway and inhibited invasion and metastasis in vitro and in vivo [53]. Further research also indicated that the knockdown of MALAT1 by siRNA significantly inhibited the migration of human osteosarcoma cell lines [59]. Mechanistically, MALAT1 increases the protein levels of RhoA and its downstream effectors namely ROCKs [59]. In human osteosarcoma tissue, HNF1A-AS1 was significantly over-expressed [72]. Downregulating HNF1A-AS1 prevented metastasis and influenced the Wnt/β-catenin pathway activity, indicating that HNF1A-AS1 promotes metastasis in osteosarcoma via the Wnt/β-catenin pathway [72]. BCAR4 was significantly upregulated in human osteosarcoma tissue [73]. Knockdown of BCAR4 inhibited the metastasis of human osteosarcoma cell lines in vitro and in vivo [73]. Mechanistically, BCAR4 promotes osteosarcoma cell metastasis by activating the GLI2-dependent pathway [73] (Table 2).

Some lncRNAs regulate various RNAs, such as miRs and lncRNAs. The plasmacytoma variant translocation 1 (PVT1) was over-expressed in human osteosarcoma tissue [66]. Inhibiting PVT1 reduced miR-195 activity and subsequently depressed osteosarcoma cell ability for migration and invasion [66]. The inhibition of PVT1 had a cascading effect, in that it impeded miR-195 activity that then blocked fatty acid synthase (FASN), and, overall, the invasiveness of human osteosarcoma cell lines consequently decreased [66]. Moreover, ZEB1-AS1 expression was upregulated in human osteosarcoma tissue and cell lines [74]. The overexpression of ZEB1-AS1 was correlated with tumor metastasis in osteosarcoma patients [74]. By contrast, ZEB1-AS1 knockdown inhibited osteosarcoma cell migration [74]. Silencing ZEB1 prevented ZEB1-AS1 from inducing osteosarcoma cell migration [74] (Table 2).

Additionally, lncRNAs execute function through various mechanisms to promote osteosarcoma cell metastasis, such as influencing gene expression. For example, the overexpression of hypoxia-inducible factor-2α (HIF-2α) lncRNA promoter upstream transcript (HIF2PUT) decreased cell migration and self-renewal in human osteosarcoma cell lines, and predictably the knockdown of HIF2PUT had the opposite effect [57]. HIF2PUT may act partly by controlling HIF-2α expression [57]. In osteosarcoma samples, HOTAIR was commonly over-expressed [60]. The inhibition of HOTAIR significantly prevented invasion and metastasis, an observation whose mechanism may lie in the association between MMP2, MMP9 and HOTAIR [60]. Another study showed that antisense non-coding RNA in the INK4 locus (ANRIL) expression was upregulated in human osteosarcoma tissue when compared with the surrounding normal tissue [82]. In vitro, hypoxia can upregulate ANRIL expression through direct coalition between hypoxia inducible factor 1α (HIF-1α) and the putative hypoxia response element located upstream of the ANRIL gene; subsequently, ANRIL promoted cancer cell invasion and suppressed apoptosis [82]. Additionally, under hypoxic conditions, HIF-1α suppression downregulated ANRIL [82] (Table 2).

### 3.4. LncRNAs in Osteosarcoma Chemoresistance

Neoadjuvant chemotherapy is a critical component of osteosarcoma treatment yet, if chemoresistance occurs, it impedes clinical efficacy. Recently, a variety of lncRNAs with multiple biological functions have been linked with chemoresistance in osteosarcoma. Two research avenues may provide insight into novel approaches to overcome drug resistance in osteosarcoma, namely: investigating lncRNA expression changes in multi-drug resistant cells and their drug-sensitive counterparts; and, secondly, analyzing differentially expressed lncRNAs and their corresponding epigenetic modifications at the onset of chemoresistance.

A human lncRNA-mRNA combined microarray revealed the overexpression of 3465 lncRNAs (1761 upregulated and 1704 downregulated) and 3278 mRNAs (1607 upregulated and 1671 downregulated) in doxorubicin-resistant MG63 cells in comparison with their paired parental MG63 cells [83]. This result suggests a complicated interaction between lncRNAs and mRNAs [83]. After Gene Ontology and pathway analysis, an lncRNA-mRNA co-expression network for osteosarcoma was constructed and it demonstrated that a set of differentially expressed lncRNAs regulate chemoresistance through various pathways [83]. The most dysregulated lncRNAs—namely ENST00000563280 (osteosarcoma doxorubicin-resistance related upregulated lncRNA, ODRUL) and NR-036444—were critical for doxorubicin resistance due to their interactions with important genes including: ATP-binding cassette, subfamily B, member 1 (ABCB1), HIF-1α and forkhead box protein C2 (FOXC2) [83]. Furthermore, the overexpression of ODRUL was associated with a poorer chemotherapeutic response and a shorter survival time in osteosarcoma patients, which indicates its potential as a biomarker for the prediction of chemoresistance in osteosarcoma [83]. ODRUL was upregulated in doxorubicin resistant human osteosarcoma cell lines in comparison with doxorubicin sensitive human osteosarcoma cell lines [84]. ODRUL was linked with poor chemotherapeutic response in osteosarcoma patients as well as doxorubicin resistance in human osteosarcoma cell lines [84]. Mechanistically, ODRUL may increase doxorubicin resistance by inducing the expression of a classic multidrug resistance-related ABCB1 gene in human osteosarcoma cell lines [84]. In human osteosarcoma cell lines, cisplatin induced the expression of LINC00161 [85]. The overexpression of LINC00161 increased cisplatin-induced apoptosis and reversed cisplatin resistance in human osteosarcoma cell lines [85]. Expectedly, LINC00161 inhibition contributed to cisplatin resistance in human osteosarcoma cell lines [85]. Mechanistically, LINC00161 can sequester endogenous miR-645 and inhibit its activity, resulting in the upregulation of the interferon-induced protein with tetratricopeptide repeats 2 (IFIT2) [85]. HOXA Distal Transcript Antisense RNA (HOTTIP) was detected at heightened concentrations in human osteosarcoma tissue [86]. The overexpression of HOTTIP was correlated with chemoresistance in human osteosarcoma cell lines [86]. Further mechanistic studies showed that HOTTIP induced cisplatin resistance via activation of the Wnt/β-catenin pathway [86].

### 3.5. LncRNAs in Osteosarcoma Prognosis

Nowadays, molecular biological studies in osteosarcoma have not contributed to the clinical realization of prognostic stratification [75]. Some prognostic information may be derived from the loss of tumor suppressor genes, such as p53 [75]. More recently, increasing studies indicate that lncRNAs can be effective prognostic biomarkers in osteosarcoma patients.

When compared with normal controls, highly upregulated in liver cancer RNA (HULC) was significantly upregulated in human osteosarcoma tissue and cell lines, as well as correlated with a shorter overall survival in osteosarcoma patients [81]. Another study demonstrated that the upregulation of HULC was associated with overall and event-free survival rates in osteosarcoma patients, suggesting HULC’s potential as a prognostic biomarker [87]. HOTTIP expression was high in human osteosarcoma tissue [62]. Osteosarcoma patients with elevated HOTTIP expression had poorer overall survival than those with low HOTTIP expression [62]. Multivariate Cox proportional hazards regression analysis revealed HOTTIP expression to be an independent prognostic factor of overall survival in osteosarcoma patients [62]. The expression of UCA1 was elevated in human osteosarcoma tissue and cell lines, and was correlated with poor prognosis [69]. Multivariate regression analysis further suggested UCA1 overexpression to be an independent negative prognostic factor [69]. Recent studies have sparked a debate regarding the previous understanding of the function of HIF2PUT in osteosarcoma. Compared with normal bone tissue, the expression of HIF2PUT was significantly upregulated in human osteosarcoma tissue [88]. The overexpression of HIF2PUT in osteosarcoma patients was positively correlated with shorter overall and disease-free survival [88]. Through Cox multivariate analysis, HIF2PUT expression was one of the independent and significant prognostic factors for both overall and disease-free survival [88]. MALAT1 was significantly upregulated in human osteosarcoma tissue compared with paired non-tumor tissue [89]. Kaplan–Meier survival analysis and multivariate Cox regression analysis revealed MALAT1 expression to be an independent prognostic factor for the shorter overall survival rate observed in osteosarcoma patients [89]. TUG1 was over-expressed in human osteosarcoma tissue in comparison with matched adjacent normal tissue [78]. The upregulation of TUG1 was strongly correlated with a poor prognosis and was an independent prognostic indicator for overall survival and progression-free survival [78]. In blood samples of osteosarcoma patients, TUG1 expression levels were decreased in postoperative patients in comparison with preoperative patients, and the changes of TUG1 expression were significantly associated with disease status [78]. Moreover, TUG1 expression was successful at distinguishing osteosarcoma patients from healthy individuals [78]. The expression of 91H, which is the H19 antisense RNA, was notably increased in both osteosarcoma patients and cell lines when compared to healthy controls and normal human bone cell lines [90]. High expression of 91H was significantly correlated with advanced clinical stage, postoperative chemotherapy, and large tumor size [90]. Furthermore, 91H expression was shown to be an independent prognostic factor for overall survival in osteosarcoma patients after treatments [90]. In addition, several other lncRNAs, including BCAR4, FGFR3-AS1, HNF1A-AS1 and ZEB1-AS1 were highly expressed in human osteosarcoma patients and correlated with poor prognosis [65,72,73,74] (Table 3).

In contrast, when compared with adjacent normal tissue, some lncRNAs—such as MEG3 and TUS7—were downregulated in human osteosarcoma tissue [68,91]. The overexpression of MEG3 or TUSC7 was associated with longer overall survival [68,91]. Multivariate analysis indicated the expression of MEG3 to be one independent predictor of overall survival in osteosarcoma patients [91] (Table 3).

## 4. The Functions and Mechanisms of LncRNAs in Other Sarcomas

In addition to osteosatcoma, deregulations of lncRNAs have been observed in chondrosarcoma, Ewing’s sarcoma, gastrointestinal stromal tumors (GISTs) and liposarcoma.

### 4.1. LncRNAs in Chondrosarcoma

Chondrosarcoma is the second most common primary bone malignancy. Chondrosarcoma is a heterogeneous collection of cartilaginous-origin tumors with different outcomes depending on subtype and histological grade. Unlike many tumors, chondrosarcoma is usually resistant to chemotherapy and radiotherapy.

The expression of HOTAIR was upregulated in chondrosarcoma tissues and cell lines [92]. High HOTAIR expression was verified correlated with tumor stage and poor prognosis [92]. Knockdown of HOTAIR led to growth inhibition of human chondrosarcoma cells in vitro and in vivo [92]. Downregulation of HOTAIR resulted in growth inhibition of human chondrosarcoma cells via G0/G1 arrest and apoptosis. Meanwhile, suppression of HOTAIR inhibited autophagy [92]. Mechanistically, HOTAIR induces DNA methylation of miR-454-3p by recruiting enhancer of zeste homolog 2 (EZH2) and DNA methyctransferace 1 (DNMT1) to the miR-454-3p promoter regions, which markedly silences miR-454-3p expression [92]. Furthermore, signal transducer and activator of transcription 3 (STAT3) and autophagy-related gene 12 (*ATG12*) are targets of miR-454-3p, initiate HOTAIR deficiency-induced apoptosis and reduce autophagy [92].

### 4.2. LncRNAs in Ewing’s Sarcoma

Ewing’s sarcoma is a highly aggressive and metastatic tumor in children and young adults. The standard treatment of Ewing’s sarcoma includes chemotherapy, surgery and radiation. Although the five-year survival rate for primary Ewing’s sarcoma has improved, the survival rate remains low for patients with metastases or recurrence [93]. Emerging studies suggest that lncRNAs contribute to cell proliferation in Ewing’s sarcoma. The chromosomal fusion between the Ewing sarcoma breakpoint region 1 (EWSR1) gene and the transcription factor friend leukemia virus integration site 1 (FLI1) gene is found in 85% of these tumors [93]. In addition to FLI1, fusions can be found between EWSR1 and other E-twenty six (ETS) family transcription factors [93].

EWSAT1 was found to be induced and upregulated by EWS-FLI1 in primary pediatric human mesenchymal progenitor cells [94]. The inhibition of EWSAT1 expression diminished cell viability in human Ewing sarcoma cell lines [94]. The co-expression of EWS-FLI1 and EWSAT1 demonstrated their synergistic role in repressing gene expression [94]. Upon further RNA sequencing analysis from primary human Ewing sarcoma tissue, EWSAT1 was found to be critical for gene repression downstream of EWS-FLI [94]. The repressive function of EWSAT1 was mediated in part by its interaction with an RNA-binding protein called heterogeneous nuclear ribonucleoprotein [94].

### 4.3. LncRNAs in GISTs

GISTs are mesenchymal neoplasms of the gastrointestinal tract and they represent 1% of gastrointestinal malignancies [95]. The outcome of patients with GISTs has significantly improved due to two primary innovations, namely the discovery of a mutation in the receptor tyrosine kinase (RTK) KIT proto-oncogene receptor tyrosine kinase (KIT) and pharmacological treatment with Gleevec (Imatinib mesylate) [96]. Gleevec was developed for Ph-positive (Philadelphia chromosome) chronic myeloid leukemia patients, and it targets the protein product from the trademark translocation between chromosomes 9 and 22 [97]. Gleevec targets a particular subset of protein tyrosine kinases in addition to any other known oncogenic isoform [97]. Therefore, Gleevec was extended to treat GISTs after its therapeutic potency was demonstrated [98]. However, some patients still develop recurrent or metastatic disease after treatment [99]. Recently, studies suggest that lncRNAs may influence metastasis in GISTs.

The HOTAIR gene was upregulated in GISTs with miR-196a overexpression, which suggests that HOTAIR may have a synergistic role alongside miR-196a [100]. The upregulation of HOTAIR was associated with metastasis and a high-risk grade [100]. RNA interference-mediated knockdown of HOTAIR altered the expression of HOTAIR target genes and suppressed GIST cell invasiveness [100].

### 4.4. LncRNAs in Liposarcoma

Liposarcoma is the most common soft tissue sarcomas, accounting for roughly 20% of sarcomas. Patients with high-grade or unresectable liposarcoma have poor prognosis, although surgery and some drugs including doxorubicin, ifosfamide, antimitotic docetaxel and antimetabolites gemcitabine seem helpful [101,102].

LncRNA Proliferation Interacting LncRNA in Retroperitoneal Liposarcoma (PILRLS) was over-expressed in retroperitoneal liposarcoma [103]. The knockdown of PILRLS significantly inhibited cell proliferation and colony formation of etroperitoneal liposarcoma cells [103]. The RNA pull-down assay revealed that PILRLS specifically bound to the T-cell leukemia 1A protein (TCL1A) [103]. Moreover, the binding between PILRLS and TCL1A suppressed the P53 pathway and activated the expression of the murine double mimute 2 (MDM2) and protein kinase B (AKT), which caused unlimited growth of retroperitoneal Liposarcoma cells [103].

## 5. Conclusions

With the development of advanced molecular biological techniques—particularly microarray and high-throughput screening—growing evidence demonstrates that lncRNAs are involved in sarcomas, namely osteosarocma, Ewing’s sarcoma and GISTs. LncRNAs are now known to affect sarcoma cell proliferation, metastasis and drug resistance through different mechanisms including: influencing specific pathways, interacting with targeted miRs or lncRNAs, regulating gene expression, and more. However, they also can enhance the sensitivity of diagnostics and prognostics in sarcoma patients. Current emerging data emphasizes the potential that lncRNAs have as biomarkers to enable more informative clinical management of sarcoma. In addition, lncRNAs are attractive targets for the development of sarcoma therapeutic modalities due to their cell type and unique expression profiles.

However, there is still more research regarding lncRNAs to be done, including the topics described in the following list: (1) although some sarcoma related lncRNAs have been discovered, there are many more unknown lncRNAs; (2) most lncRNA related-research focuses on osteosarcoma; therefore, more research on other sarcoma types is needed; (3) the underlying mechanisms of known lncRNAs that influence sarcoma phenotypes, such as HOTAIR or MALAT1, require further investigation; (4) one lncRNA can be linked with multiple phenotypes of the same sarcoma, which raises questions related to that particular lncRNA’s role in pathogenesis; HIF2PUT in osteosarcoma is an example; (5) lastly, in sarcoma, lncRNAs—namely HOTAIR, LINC00161, PVT1 and TUG1—were found to interact as ceRNAs with miRNAs. Although a phenomenon observed in previous studies, an enriched understanding of the relationship between lncRNAs and other transcriptional factors is still needed [104].

In the near future, the development of experimental tools and technologies related to long-read sequencing can enable the identification and analysis of lncRNA mechanisms. Moreover, targeted deletion studies in primary cells and animal models will be necessary to assess the specific functions of lncRNAs in sarcoma. Although the functions and underlying mechanisms of various lncRNAs are still far from fully understood, future studies on lncRNA-mediated tumorigenesis in sarcoma can verify lncRNA candidacy for the innovation of early diagnostic, treatment and prognostic prediction approaches.

## Figures and Tables

**Table 1:** Long non-coding RNAs associated with cell proliferation of osteosarcoma.

LncRNA Name	Expression in OS	OS Cells	Potential Mechanism	Tumor Cell Proliferation	Ref.
TUG1	High	U2OS cells	Inhibit apoptosis	Promote	[55]
		U2OS and Saos-2 cells	TUG1/miR-9-5p/ POU2F1 pathway		[56]
H19	High	Kios5 cells	Hh signaling pathway and the oncogene Yap1	Promote	[52]
HIF2PUT	Low	SAOS2, MG63, U2OS and OS-732 cells	Control HIF-2α expression	Inhibit	[57]
BANCR	Low	MG63 cells	JNK and Wnt/β-catenin signaling pathway	Inhibit	[54]
MALAT1	High	U2OS and Saos2 cells	PI3K/AKT signaling pathway	Promote	[53]
		Saos2, MG63, U2OS cells	Target TGFA promotion via Inhibiting MIR376A		[58]
		U2OS, HOS, 143B and MG63 cells	Increase RhoA and its downstream effectors ROCKs		[59]
HOTAIR	High	U2OS, HOS, 143B and MG63 cells	Activate MMP2 and MMP9	Promote	[60]
		MG63 cells	Upregulate TGF-β and Bcl-2, upregulate p53 and TNF-α		[61]
HOTTIP	High	MG-63 and HOS cells	-	Promote	[62]
ANCR	High	U2OS and Saos2 cells	Related to p21,CDK2	Promote	[63]
EWSAT1	High	MG63 and HOS cells	Inhibit MEG3 expression	Promote	[64]
FGFR3-AS1	High	MG63 and U2OS cells	Upregulate FGFR3 expression	Promote	[65]
PVT1	High	KHOS, 143b, LM7, U2OS, and MG63 cells	Increase BCL2 and CCND1 protein expression via negatively regulating miR-195	Promote	[66]
SNHG12	High	Saos-2, MG63 and U2OS cells	Upregulate AMOT mRNA expression	Promote	[67]
TUSC7	Low	HOS and MG63	Promote apoptosis	Inhibit	[68]
UCA1	High	HOS, Saos-2, MG63, U2OS cells	Inhibit apoptosis	Promote	[69]
MFI2	High	SAOS-2, MG63, and U2OS cells	Upregulate FOXP4	Promote	[70]
PACER	High	143B, MG63, Saos-2, U2OS cells	Activate NF-κB-dependent COX-2	Promote	[71]
HNF1A-AS1	High	HOS, SaOS2, MG63 and U2OS cells	Wnt/β-catenin pathway	Promote	[72]
BCAR4	High	MG63 and U2OS cells	Activate GLI2 pathway	Promote	[73]
ZEB1-AS1	High	HOS, U2OS, MG-63, and Saos-2 cells	Related to ZEB1 lncRNA	Promote	[74]

OS, osteosarcoma; TUG1, taurine upregulated gene 1; Chr, chromosome; Yap1, yes-associated protein 1; Hh, Hedgehog; HIF2PUT, hypoxia-inducible factor-2α (HIF-2α) promoter upstream transcript; BANCR, BRAF-regulated lncRNA 1; JNK, c-Jun N-terminal kinases; MALAT1, Metastasis-associated lung adenocarcinoma transcript 1; HOTAIR, homeobox (HOX) transcript antisense RNA; MMP, matrix metalloproteinase; ANCR, antidifferentiation noncoding RNA; PI3K, phosphatidylinositol-3-kinase; AKT, protein kinase B; EWSAT1, Ewing sarcoma–associated transcript 1; FGFR3-AS1, FGFR3 antisense transcript 1; PVT1, plasmacytoma variant translocation 1; SNHG12, small nucleolar RNA host gene 12; TUSC7, tumor suppressor candidate 7; UCA1, urothelial carcinoma associated 1; FOXP4, forkhead box P4; PACER, P50-associated COX-2 extragenic RNA; TGF-β, transforming growth factor-β; TNF-α, tumor necrosis factor-α; HNF1A-AS1, HNF1A-antisense 1; BCAR4, breast cancer anti-estrogen resistance 4; HOTTIP, HOXA Distal Transcript Antisense RNA; ROCKs, Rho-associated coiled-coil containing protein kinases; ZEB1-AS1, Zinc Finger E-Box Binding Homeobox 1 Antisense RNA 1; Ref, reference.

**Table 2:** Long non-coding RNAs associated with metastasis of osteosarcoma.

LncRNA Name	Expression in OS	OS Cells	Potential Mechanism	Metastasis	Ref.
HIF2PUT	Low	SAOS2, MG63, U2OS and OS-732 cells	Control HIF-2α expression	Inhibit	[57]
MALAT1	High	U2OS and SaO2 cells	PI3K/AKT signaling pathway	Promotes	[53]
		U2OS, HOS, 143B and MG63 cells	Increase RhoA and its downstream effectors ROCKs		[59]
HOTAIR	High	U2OS, HOS, 143B and MG63 cells	Activate MMP2 and MMP9	Promotes	[60]
HULC	High	MG-63, U2OS and Saos-2 cells	-	Promotes	[81]
HOTTIP	High	MG-63 and HOS cells	-	Promotes	[62]
ANRIL	High	HOS and U2OS cells	Activated by HIF-1α	Promotes	[82]
EWSAT1	High	MG63 and HOS cells	Inhibit MEG3 expression	Promotes	[64]
PVT1	High	KHOS, 143b, LM7, U2OS, and MG-63 cells	Increase FASN protein expression via negatively regulating miR-195	Promotes	[66]
SNHG12	High	Saos-2, MG-63 and U2OS cells	Upregulate AMOT mRNA expression	Promotes	[67]
UCA1	High	HOS, Saos-2, MG-63, U2OS cells	-	Promotes	[69]
MFI2	High	SAOS-2, MG63, and U2OS cells	Upregulate FOXP4	Promotes	[70]
PACER	High	143B, MG63, Saos-2, U2OS cells	Activate NF-κB-dependent COX-2	Promotes	[71]
HNF1A-AS1	High	HOS, SaOS2, MG63 and U2OS cells	Wnt/β-catenin pathway	Promotes	[72]
BCAR4	High	MG63 and U2OS cells	Activate GLI2 pathway	Promotes	[73]
ZEB1-AS1	High	HOS, U2OS, MG-63, and Saos-2 cells	Related to ZEB1 lncRNA	Promotes	[74]

OS, osteosarcoma; HIF2PUT, hypoxia-inducible factor-2α (HIF-2α) promoter upstream transcript; MALAT1, Metastasis-associated lung adenocarcinoma transcript 1; Chr, chromosome; HIF-1α, hypoxia-inducible factor-1α; PI3K, phosphatidylinositol-3-kinase; AKT, protein kinase B; HOTAIR, HOX transcript antisense RNA; MMP, matrix metalloproteinase; ANRIL, antisense non-coding RNA in the INK4 locus; HIF-1α, hypoxia-inducible factor-1α; PVT1, plasmacytoma variant translocation 1; SNHG12, small nucleolar RNA host gene 12; UCA1, urothelial carcinoma associated 1; FOXP4, forkhead box P4; PACER, P50-associated COX-2 extragenic RNA; HNF1A-AS1, HNF1A-antisense 1; HULC, highly upregulated in liver cancer RNA; BCAR4, breast cancer anti-estrogen resistance 4; HOTTIP, HOXA Distal Transcript Antisense RNA; ROCKs Rho-associated coiled-coil containing protein kinases; ZEB1-AS1, Zinc Finger E-Box Binding Homeobox 1 Antisense RNA 1; Ref, reference.

**Table 3:** Long non-coding RNAs associated with prognosis of osteosarcoma.

LncRNA Name	Number of OS Sample	Expression Pattern	Prognosis	Ref.
HULC	78 tissue samples	High	Poor	[81]
	33 tissue samples			[87]
MEG3	64 tissue samples	Low	Good	[91]
HOTTIP	68 tissue samples	High	Poor	[62]
UCA1	135 tissue samples	High	Poor	[69]
HIF2PUT	82 tissue samples	High	Poor	[88]
BCAR4	60 tissue samples	High	Poor	[73]
MALAT1	162 tissue samples	High	Poor	[89]
TUG1	76 tissue samples 29 blood samples	High	Poor	[78]
FGFR3-AS1	62 tissue samples	High	Poor	[65]
TUSC7	82 tissue samples	Low	Good	[68]
HNF1A-AS1	43 tissue samples	High	Poor	[72]
91H	67 tissue samples	High	Poor	[90]
ZEB1-AS1	50 tissue samples	High	Poor	[74]

LncRNA, long noncoding RNA; OS, osteosarcoma; HULC, highly upregulated in liver cancer RNA; UCA1, urothelial carcinoma associated 1; HIF2PUT, hypoxia-inducible factor-2α (HIF-2α) promoter upstream transcript; BCAR4, breast cancer anti-estrogen resistance 4; HOTTIP, HOXA Distal Transcript Antisense RNA; FGFR3-AS1, FGFR3 antisense transcript 1; TUSC7, tumor suppressor candidate 7; HNF1A-AS1, HNF1A-antisense 1; ZEB1-AS1, Zinc Finger E-Box Binding Homeobox 1 Antisense RNA 1; Ref, reference.
